# Risk Factors for Pulmonary Air Leak and Clinical Prognosis in Patients With COVID-19 Related Acute Respiratory Failure: A Retrospective Matched Control Study

**DOI:** 10.3389/fmed.2022.848639

**Published:** 2022-03-31

**Authors:** Roberto Tonelli, Giulia Bruzzi, Linda Manicardi, Luca Tabbì, Riccardo Fantini, Ivana Castaniere, Dario Andrisani, Filippo Gozzi, Maria Rosaria Pellegrino, Fabiana Trentacosti, Lorenzo Dall’Ara, Stefano Busani, Erica Franceschini, Serena Baroncini, Gianrocco Manco, Marianna Meschiari, Cristina Mussini, Massimo Girardis, Bianca Beghè, Alessandro Marchioni, Enrico Clini

**Affiliations:** ^1^Respiratory Diseases Unit, Department of Medical and Surgical Sciences, University Hospital of Modena, University of Modena Reggio Emilia, Modena, Italy; ^2^Clinical and Experimental Medicine Ph.D. Program, University of Modena and Reggio Emilia, Modena, Italy; ^3^Intensive Care Unit, University Hospital of Modena, Modena, Italy; ^4^Infectious Diseases Unit, University Hospital of Modena, Modena, Italy; ^5^Department of Surgery, University of Modena and Reggio Emilia, Modena, Italy

**Keywords:** acute respiratory failure, inspiratory effort, esophageal manometry, non-invasive mechanical ventilation, COVID-19, pneumothorax, pneumomediastinum, pulmonary air leak

## Abstract

**Background:**

The role of excessive inspiratory effort in promoting alveolar and pleural rupture resulting in air leak (AL) in patients with SARS-CoV-2 induced acute respiratory failure (ARF) while on spontaneous breathing is undetermined.

**Methods:**

Among all patients with COVID-19 related ARF admitted to a respiratory intensive care unit (RICU) and receiving non-invasive respiratory support, those developing an AL were and matched 1:1 [by means of PaO2/FiO2 ratio, age, body mass index-BMI and subsequent organ failure assessment (SOFA)] with a comparable population who did not (NAL group). Esophageal pressure (ΔP_es_) and dynamic transpulmonary pressure (ΔP_L_) swings were compared between groups. Risk factors affecting AL onset were evaluated. The composite outcome of ventilator-free-days (VFD) at day 28 (including ETI, mortality, tracheostomy) was compared between groups.

**Results:**

Air leak and NAL groups (*n* = 28) showed similar ΔP_es_, whereas AL had higher ΔP_L_ (20 [16–21] and 17 [11–20], *p* = 0.01, respectively). Higher ΔP_L_ (OR = 1.5 95%CI[1–1.8], *p* = 0.01), positive end-expiratory pressure (OR = 2.4 95%CI[1.2–5.9], *p* = 0.04) and pressure support (OR = 1.8 95%CI[1.1–3.5], *p* = 0.03), D-dimer on admission (OR = 2.1 95%CI[1.3–9.8], *p* = 0.03), and features suggestive of consolidation on computed tomography scan (OR = 3.8 95%CI[1.1–15], *p* = 0.04) were all significantly associated with AL. A lower VFD score resulted in a higher risk (HR = 3.7 95%CI [1.2–11.3], *p* = 0.01) in the AL group compared with NAL. RICU stay and 90-day mortality were also higher in the AL group compared with NAL.

**Conclusion:**

In spontaneously breathing patients with COVID-19 related ARF, higher levels of ΔP_L_, blood D-dimer, NIV delivery pressures and a consolidative lung pattern were associated with AL onset.

## Introduction

The Severe Acute Respiratory Syndrome related to Coronavirus-2 (SARS-CoV-2) infection (COVID-19) has been spreading worldwide in successive surges since end of 2019 with a critical burden of patients developing acute respiratory failure (ARF) and needing invasive (MV) and non-invasive respiratory support (NRS) (1). The associated literature has reported that the onset of pneumothorax (PNX) and pneumomediastinum (PNM), namely air leak (AL), is not an uncommon complication of COVID-19 related pneumonia (2–7) leading to worse gas exchange and increased mortality (8). A retrospective analysis reported a 13% incidence rate of PNX in mechanically ventilated patients with COVID-19 (8), twice as high as those described in typical acute respiratory distress syndrome (ARDS), where protective ventilation strategies have significantly reduced the risk of barotrauma (9, 10). Furthermore, a high incidence of PNX and PNM has been reported in COVID-19 patients even during spontaneous breathing (11–14), thus questioning the physiopathological mechanisms beyond the rupture of the alveolar and pleural walls. Some authors have hypothesized a blunt breakdown of alveolar spaces due to interstitial inflammation or vascular damage leading to dissection and air leak along the bronchoalveolar sheath into the mediastinum (*Macklin effect*) (3, 15, 16). A recent computational study in COVID-19 patients under intense inspiratory effort (i.e., a high pleural pressure swing) has shown that the physical forces produced were similar to those measured over ventilatory-induced lung injury (VILI) (17). Therefore, it might be hypothesized that both patient’s effort and lung stress play a role in favoring alveolar injury during spontaneous or assisted breathing. Nonetheless, knowledge of the factors and physiological variables associated with the development of PNX and PNM under spontaneous breathing in patients with COVID-19 are still scarce.

The purpose of this retrospective study is to investigate the role of inspiratory effort and the risk factors associated with the onset of AL in spontaneously breathing COVID-19 patients with ARF.

## Materials and Methods

### Study Setting and Design

This retrospective single center cohort study was carried out at the Respiratory Intensive Care Unit (RICU) of the University Hospitals of Modena (Italy) and conducted in accordance with the pre-existing Ethics Committee “Area Vasta Emilia Nord” approval (registered protocol number 4485/C.E., document 266/16). Informed consent to participate in the study and to allow their clinical data to be analyzed and published were obtained from participants; the study represents a retrospective sub-analysis of data prospectively collected within a pre-registered clinical trial ClinicalTrial.gov (NCT03826797).

### Patient Selection, Case Definition, and Matching

All patients with COVID-19 induced ARF admitted to our RICU receiving non-invasive respiratory support (high flow oxygen-HFNC and/or non-invasive ventilation-NIV) over the period October 1, 2020 to May 15, 2021 were retrospectively considered eligible for enrollment.

Inclusion criteria were documented SARS-CoV-2 positive real-time-polymerase chain reaction (RT–PCR) on a nasal or pharyngeal swab; onset of PNX and/or PNM (i.e., AL) while spontaneously breathing and confirmed by chest-computed tomography (CT) scan; inspiratory effort assessment and monitoring by means of esophageal manometry.

Those patients who received MV or NIV before RICU admission with an established diagnosis of chronic obstructive pulmonary disease, neuromuscular disease or chest wall deformities, interstitial lung disease or those lacking core data (i.e., clinical characteristics at baseline, physiological measurement, type, and time of outcomes) at medical record analysis were excluded.

The AL group was matched 1:1 with a COVID-19 induced ARF population with no history of AL (*n* = 333). The eligibility criteria of this latter group were based on PaO2/FiO2 ratio, age, body mass index (BMI) and sequential organ failure assessment score (SOFA), besides available esophageal manometry data (*n* = 86). All patients enrolled in the study were admitted into Hospital over the same time period.

The values of PaO2/FiO2 ratio and SOFA used for matching these groups were measured on admission, with all patients receiving HFNC in the appropriate setting. The logit of the score was taken with a caliper of 0.2 without replacement in order to maximize the number of patients without comprising the match.

### Treatments

Pharmacological therapy was in agreement with the Italian Society of Infectious Diseases’ Guidelines (SIMIT) (18) and in accordance with the evolving recommendations provided by the World Health Organization on COVID-19 pandemic^1^.

Non-invasive respiratory support (NRS) devices were adopted according to our local protocol. Pharmacological sedation while on NRS was allowed to achieve a Richmond Agitation Sedation Scale (RASS) score within the range –1 to 0. The ventilator settings of each device were adjusted by the attending physician based on continuous cardiopulmonary monitoring. These included:

– HFNC, (Optiflow™ and AIRVO™, Fisher & Paykel Healthcare Ltd., Auckland, New Zealand) delivering humidified oxygen through a nasal cannula. Flow delivery was initially set at 60 L/min and temperature at 37°C and then adjusted according to the patient’s tolerance.
– NIV, with patients connected *via* a conventional circuit with an appropriately sized oronasal facemask equipped with a dedicated output for probes (Bluestar™, KOO Medical Equipment, Shanghai, China) to a high-performance ventilator (GE Healthcare Engstrom Carestation™, GE Healthcare, Finland) in pressure support pre-set mode. PEEP was initially set at 8 cmH_2_O and subsequently fine-tuned to target a peripheral oxygen saturation (SpO_2_) > 92% with a delivered inspiratory fraction of oxygen (FiO_2_) of less than 0.7. Pressure support (PS) was set at 10 cmH_2_O, and then progressively modified depending on tidal volume to target a Vte/kg of PBW < 9.5 mL/kg and a respiratory rate < 30 breaths/min. The inspiratory trigger was set at 3 L/min and expiratory cycling was set at 25% of the inspiratory peak flow. The delivered FiO_2_ was increased to target a SpO_2_ of 88–94%. The oronasal facemask was tightened to target a leak flow lower than 20 L/min.

The criteria for being referred to RICU to upgrade to HFNC included a peripheral oxygen saturation (SpO_2_) < 90% during conventional oxygen therapy with Venturi mask and/or the presence of respiratory rate (RR) > 25 breaths/m(bpm) and/or the presence of subjective respiratory distress. In case of failure of HFNC, patients received a trial of escalation to NIV if deemed indicated by the treating clinician, blinded to the study purposes and physiological measurements. The criteria to upgrade to NIV were according to local protocols and included PaO_2_/FiO_2_ ratio 100 mmHg and/or RR > 25 bpm and/or persistence of respiratory distress and dyspnea despite HFNC set at 60 L/min. NIV was delivered continuously on days 1–2, then as long as possible or according to the clinical judgment with HFNC used during time in-between NIV sessions. The decision as whether to proceed to ETI was taken according to the best clinical practice by the attending staff blinded to the study purposes and physiological measurements. Criteria for ETI included: (a) PaO2/FiO2 ratio unchanged or worsened despite the use of NRS, (b) the need to protect the airway due to neurological deterioration or from a large amount of secretions, (c) hemodynamic instability or major electrocardiographic abnormalities, and (d) unchanged or worsened dyspnea and persistence of respiratory distress (i.e., respiratory rate-RR > 35 bpm, gasping for air, psychomotor agitation requiring sedation, abdominal paradox) despite NRS.

### Physiological Measurements

Esophageal pressure swing (ΔP_es_) was assessed through a multifunctional nasogastric tube with a pressure transducer (NutriVent™, SIDAM, Mirandola, Italy) connected to a dedicated monitoring system (OptiVent™, SIDAM, Mirandola, Italy) and following the recommended calibration protocol (19). For all measurements, the start of the inspiratory phase was identified at the point of P_es_ initial decline while the end of inspiration was considered to be the value of P_es_ where 25% of the time had elapsed from maximum deflection to the baseline (Supplementary Figure 1). ΔP_es_ was calculated as the negative deflection of P_es_ from the onset of inspiratory effort. Dynamic transpulmonary pressure (ΔP_L_) was defined as the tidal change in transpulmonary pressure, calculated as airway pressure (P_*aw*_) minus P_es_ (19). ΔP_es_ and ΔP_L_ were recorded daily in each patient over 3 consecutive minutes of stable spontaneous breathing in accordance with the local protocol. Esophageal manometry was performed in all patients upgrading to NRS who gave informed consent to the procedure. For patients receiving NIV treatment ΔP_es_ and ΔP_L_ were always recorded while on NIV. The attending physicians were blinded to the results of physiological measurements.

### Covariate Variables

Chart review over the medical record and archived data collection was performed. The relevant variables were then inserted into an electronic database.

Demographics, comorbidities (by Charlson index), functional characteristics (PaO2/FiO2 ratio, RR, mean arterial pressure-MAP), laboratory tests (blood lactate, C-reactive protein-CRP, and D-dimer levels), were reported on admission. Lung patterns (interstitial or consolidative) on the computed tomography scan were evaluated by a radiologist with expertise in chest CT scan and blinded to the purpose of the study. A qualitative assessment was performed; in case of co-existence of both interstitial and consolidative pattern the most represented was considered.

ΔP_es_ and ΔP_L_ considered for analysis were the average values of those measured over 3 days before the onset of air leak in AL group, while they were average values of those measured over 3 days before the highest recorded ones in NAL group. For patients receiving HFNC, PEEP was calculated as 1 cmH2O each 10 L/min with patient keeping mouth closed (20). For these patients we considered ΔP_es_ as equal to ΔP_L_.

Finally, the type of NRS required during RICU stay, the pressure delivered by the ventilator while on NIV, clinical outcomes (i.e., need for chest drainage, ETI, tracheostomy, duration of MV, mortality, length of RICU and hospital stay) were recorded.

### Study Goals

We planned first to measure the physiological variables (namely ΔP_es_ and ΔP_L_) in the two groups and to assess the association between the development of AL and the considered covariates.

Then, the composite outcome ventilator-free-days (VFD) at day 28 (including ETI, mortality, tracheostomy) was compared between the two groups; VFD scored from 0 (if the patient died or remained on mechanical ventilation within 28 days from admission), to 28 (if the patient was alive or extubated between days 1 and 28).

Mortality rate at day 90, length of RICU and hospital stay were also described in the two groups.

### Statistical Analysis

Considered variables were compared in the two groups; continuous variables were expressed as median and interquartile ranges (IQR) and compared by *t*-test and Wilcoxon–Mann–Whitney test, whereas categorical variables were reported as numbers and percentages (%) and compared by χ2 test or Fisher’s exact test as appropriate.

The association between demographic, clinical, physiological, and radiological characteristics with the onset of AL was tested by means of multivariable logistic regression model. In a *post-hoc* sensitivity analysis we separated HFNC and NIV patients to explore the predictors of AL according to the type of NRS. The association of AL with other pre-specified outcomes was further carried out through Fisher’s exact test and Wilcoxon–Mann–Whitney test.

Ventilator-free-days composite outcome was assessed in a risk regression analysis according to the Fine and Gray competing risk method and considering the patient’s status as alive and extubated at day 28 as the event of interest, whereas dead or still intubated at day 28 as the competing risk. Outcome was reported as sub-distribution hazard ratio (SHR) and then illustrated by means of unweighted Kaplan-Meier curves. A two-sided test of less than 0.05 was considered statistically significant. Statistics were performed using SPSS version 25.0 (IBM Corp., New York, NY, United States) and Graphpad prism version 8.0 (Graphpad Software, Inc., La Jolla, CA, United States) unless otherwise indicated.

## Results

**FIGURE 1:**
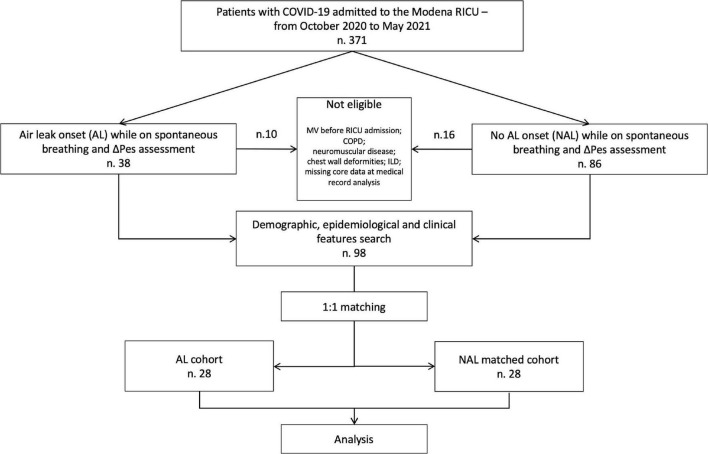
Study algorithm. COVID-19, coronavirus disease 2019; RICU, respiratory intensive care unit; AL, air leak; NAL, non-air leak; COPD; chronic obstructive pulmonary disease; ΔPes, esophageal pressure swing.

The flowchart of this study is shown in Figure 1. Over the study period a total of 371 patients were considered for enrollment.

Of these, 38 (10.1%) presented AL during RICU stay, of which 28 (7.4%) were eligible; out of them 17 (61%) developed AL while on NIV and 11 (39%) on HFNC. Eight (29%) and 6 (21%) patients developed isolated PNX and PNM, respectively, whereas 14 (50%) patients developed a combination of the two. Twelve (43%) out of these individuals required chest drainage, with 4 (14%) of them showing persistent leak (2 were successfully treated with endobronchial valve positioning). The median time from the onset of symptoms and from hospital admission to the development of AL was 23 (IQR 14 – 28) days and 18 days (IQR 11 – 25), respectively. The median time spent on NRS before developing AL was 5 days (4 – 6.3) and was longer for patients who received only HFNC (6 [5 – 7]) as compared to patients who underwent NIV treatment (4 [3.5 – 5.5]).

Matched group (*n* = 28) had similar baseline characteristics except for the median value of D-dimer that was higher in AL compared with NAL (3,340 [1,578–10,890] and 1,200 [980–1,633] μg/L, *p* = 0.02) (Table 1). No group difference was found for ΔP_es_ (13 [12–18] and 11 [10–17] cmH_2_O, *p* = 0.7, respectively), whilst AL presented higher ΔP_L_ as compared with NAL (20 [16–21] and 17 [11–20], *p* = 0.01). A larger but not significant proportion of patients in the AL group received NIV (68% and 50%, *p* = 0.2, respectively). Furthermore, the median value of PEEP (10 [8–10] and 8 [8–10] cmH_2_O, respectively, *p* = 0.01) and PSV (10 [8–12] and 8 [8–10] cmH_2_O, *p* = 0.01) was higher in AL than in NAL. The consolidative CT radiologic lung pattern was more prevalent in the AL than in the NAL group (68 and 39%, respectively, *p* = 0.03). When grouped according to NRS support, D-dimer, CRP and WBC resulted higher - within the HFNC group - in those who developed AL as compared to NAL patients (*p* < 0.0001, *p* = 0.02, *p* = 0.04, respectively) (Supplementary Table 1). Patients who developed AL under NIV presented higher ΔP_L_ (*p* = 0.02) and D-dimer (*p* = 0.04) and more elevated PEEP and PSV values (*p* = 0.01 and *p* = 0.01, respectively). The use of Tocilizumab was also less prevalent (*p* = 0.03) (Supplementary Table 1).

**TABLE 1:** General and clinical features of the study population presented as a whole and according to the onset of air leak.

Variable	Overall *n* = 56 (100)	Air leak (AL) *n* = 28 (50)	No air leak (NAL) *n* = 28 (50)	*p*-value
Age, years (IQR)	73 (61–76)	74 (60–78)	72 (61–75)	0.9
Male sex, n (%)	37 (66)	18 (64)	19 (68)	0.9
BMI, Kg/m^2^	27 (24–29)	26 (24–29)	27 (24–29)	0.9
SOFA, score (IQR)	3 (3–4)	3 (3–4)	3 (3–4)	0.9
Charlson index, score (IQR)	2 (2–4)	2 (2–4)	2 (1–5)	0.6
PaO_2_/FIO_2_, mmHg (IQR)	101 (88–114)	102 (91–114)	101 (88–119)	0.9
RR, bpm (IQR)	26 (24–29)	28 (24–30)	26 (24–29)	0.6
MAP, mmHg (IQR)	76 (68–90)	75 (65–85)	80 (68–90)	0.8
Lactate, mmol/L (IQR)	1 (0.7–1.4)	1 (0.7–1.5)	1 (0.6–1.4)	0.8
ΔP_es_, cmH_2_O (IQR)	12 (11–18)	13 (12–19)	11 (10–17)	0.7
ΔP_L_, cmH_2_O (IQR)	18 (12–20)	20 (16–21)	17 (11–20)	0.01
Time from disease onset to RICU admission, days (IQR)	9 (4–13)	10 (4–13)	8 (4–12)	0.5
**Laboratory tests**				
White cells count, n*10^9^/L (IQR)	6.5 (4.9 – 9.7)	8.2 (5.1–12.2)	5.8 (4.7–9.6)	0.7
C-Reactive Protein, mg/dL (IQR)	9.3 (6–21)	11.2 (6.5–18)	7.8 (3.6–21)	0.3
D-Dimer, μg/L (IQR)	1605 (1000 – 5295)	3340 (1578–10890)	1200 (980–1633)	0.02
**Pharmacological treatment**				
Systemic steroids, n (%)	53 (95)	27 (96)	26 (93)	0.2
Tocilizumab, n (%)	48 (86)	23 (82)	25 (89)	0.4
**Non-invasive support**				
HFNC, n (%)	23 (41)	9 (32)	14 (50)	0.2
NIV, n (%)	33 (59)	19 (68)	14 (50)	0.2
PEEP, cmH_2_O (IQR)	8 (8–10)	10 (8–10)	8 (8–10)	0.03
PSV, cmH_2_O (IQR)	10 (8–12)	10 (10–12)	8 (8–10)	0.02
NRS duration, days (IQR)	9 (3 – 21)	10 (3 – 23)	9 (3 – 21)	0.3
**Radiographic pattern**				
Interstitial, n (%)	26 (46)	9 (32)	17 (61)	0.03
Consolidative, n (%)	30 (54)	19 (68)	11 (39)	0.03

Data are presented as number and percentage for dichotomous values or median and interquartile range (IQR) for continuous values. Significance was set for p < 0.05. BMI, body mass index; IQR, inter quartile range; RR, respiratory rate; MAP, mean arterial pressure; SOFA, subsequent organ failure assessment; HFNC, high flow nasal cannula; NIV, non-invasive mechanical ventilation; PEEP, positive end expiratory pressure; PSV, pressure support; LDH, lactic dehydrogenase; ΔPes, esophageal pressure swing; and ΔP_L_, dynamic transpulmonary pressure. *Stands for multiplication sign ×.

**FIGURE 2:**
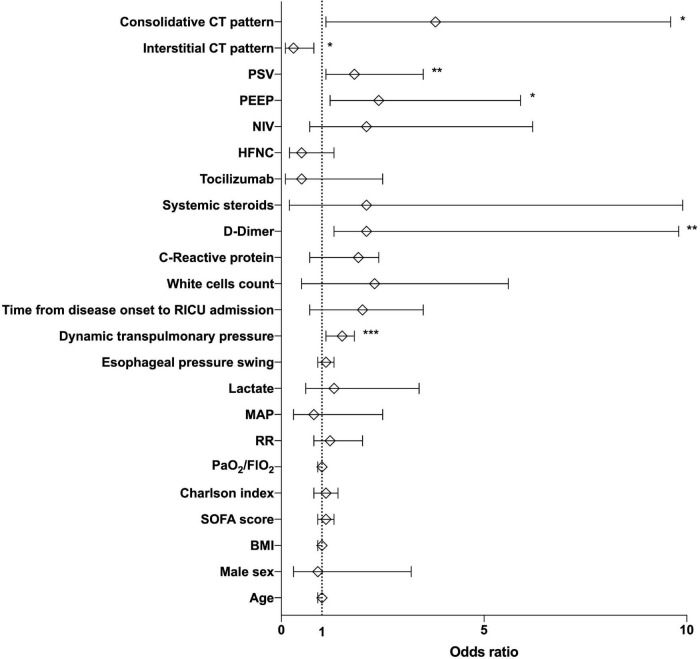
Risk factors for air leak onset while on spontaneous breathing during COVID-19 acute respiratory failure. Multiple logistic regression analysis showing the association between clinical, physiological, and radiological variables with the occurrence of pneumothorax and pneumomediastinum in the study cohort by means of odds ratios (open diamonds) and relative 95% confidence intervals (error bars). *p = 0.04, **p = 0.03, and ***p = 0.01. Significance was set for *p* < 0.05. CT, computed tomography; PSV, pressure support; PEEP, positive end-expiratory pressure; NIV, non-invasive ventilation; HFNC, high-flow nasal cannula; RICU, respiratory intensive care unit; MAP, mean arterial pressure; RR, respiratory rate; SOFA sequential organ failure assessment; BMI, body mass index.

Multiple logistic regression analysis revealed that higher ΔP_L_ (OR = 1.5 95%CI[1–1.8], *p* = 0.01), PEEP (OR = 2.4 95%CI[1.2–5.9], *p* = 0.04) and PSV (OR = 1.8 95%CI[1.1–3.5], *p* = 0.03), baseline D-dimer (OR = 2.1 95%CI[1.3–9.8], *p* = 0.03), and a consolidative lung pattern (OR = 3.8 95%CI[1.1–15], *p* = 0.04) were associated with the onset of AL (Figure 2). When separated according to NRS, patients with NIV were the only ones in whom ΔP_L_, PEEP and PSV values remained associated with AL (Supplementary Figure 1). For patients receiving only HFCN higher blood values of D-dimer and CRP were associated with AL occurrence (Supplementary Figure 2).

**FIGURE 3:**
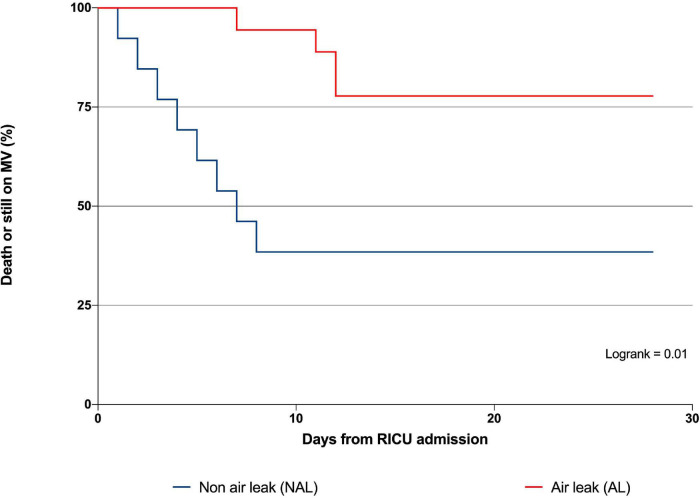
Twenty-eight-day survival analysis of patients with and without air-leak. Kaplan–Meyer analysis for composite outcome ventilator free days at day 28 in patients with and without air leak. Patients with AL presented an increased risk of being dead or still on mechanical ventilation at day 28 by 270% as compared to NAL. Significance was set for *p* < 0.05. MV, mechanical ventilation; RICU, respiratory intensive care unit; AL, air leak; NAL, non-air-leak.

VFD score was lower in AL than in NAL patients (16 IQR [0–28] and 28 IQR [21–28] days, respectively, *p* = 0.01) and resulted in a SHR of 3.7 (95%CI [1.2–11.3], *p* = 0.01) of being dead or still on MV at day 28. That is to say that an increase in the risk of the unfavorable pre-specified outcome by 270% compared to NAL. The Kaplan Meier survival curve of the two groups is showed in Figure 3.

Table 2 shows all the clinical outcomes recorded in the studied population. RICU stay (16 IQR [7–27] and 10 IQR [5–2] days, *p* = 0.01, respectively) and mortality rate at day-90 (57 and 21%, *p* = 0.01) were also higher in AL compared with NAL.

**TABLE 2:** Clinical outcomes of the study population presented as a whole and according to the onset of AL.

Outcome	Cohort			OR 95%CI	*p-value*
	
	Overall *n* = 56	Air leak *n* = 28	No air leak *n* = 28		
ETI, n (%)	31 (55)	18 (64)	13 (46)	2.1 (0.8–6.3)	0.3
Mortality at day 28, n (%)	14 (20)	10 (36)	4 (14)	3.3 (0.9–11)	0.1
Mortality at day 90, n (%)	22 (39)	16 (57)	6 (21)	4.9 (1.5–15)	0.01
Tracheostomy, n (%)	5 (9)	4 (14)	1 (4)	4.5 (0.6–57)	0.4
RICU stay, days (IQR)	13 (6–26)	16 (7–27)	10 (5–20)	–	0.01
Hospital stay days, n (%)	23 (7–45)	24 (9–45)	20 (7–41)	–	0.1

*OR, odds ratio; 95%CI, 95% confidence interval; IQR, interquartile range; and RICU, respiratory intensive care unit.*

## Discussion

Our retrospective study has shown that in a population of COVID-19 patients with severe ARF breathing spontaneously and requiring NRS, the onset of AL (i.e., PNX or PNM) was significantly associated with higher dynamic transpulmonary pressure compared with NAL, despite a similar magnitude of inspiratory effort. Higher pressures delivered during NIV, elevated D-dimer on admission and a consolidative lung pattern on CT resulted in significant risk factors for air leak in this population and demonstrated worse short- and long-term clinical outcomes.

To our knowledge this it is the first study aimed at evaluating the physiological risk factors associated with AL in a population of COVID-19 patients with ARF and breathing spontaneously. In particular, we have reported a correlation between higher dynamic transpulmonary pressure and the occurrence of PNX-PMN.

Although clinical and radiological presentation of COVID-19 related ARF is highly heterogenous, the elevated prevalence of PNX-PNM during assisted spontaneous breathing questions the physiological mechanisms under the alveolar and pleural wall rupture (2). A growing body of evidence shows that a strong inspiratory effort can lead to excessive lung strain, mirroring the risk of high tidal volume delivered by positive insufflation during mechanical ventilation (21). Some researchers have indeed hypothesized that increased respiratory effort might translate into severe intrapulmonary strain resulting in the breakdown of alveolar spaces (22). Thus, an excessive respiratory drive might be considered as a promoter of air leak during an acute lung injury.

Our patients presented an increase in inspiratory effort (median ΔP_es_ = 12 cmH_2_O), in line with recently published data on the esophageal pressure swing during the early phase of COVID-19 (23, 34). ΔP_es_ was similar among groups despite difference in the severity of the disease. These data confirm a relatively low activation of respiratory drive in patients with moderate to severe ARF due to COVID-19 during the early phase of NRS, which is in line with the clinical concept of “happy hypoxemia” (24) and underlines the mismatch between central drive activation and moderate to severe forms of lung impairment, at variance with the typical form of ARDS. Moreover, the use of sedation might have improved patient interaction receiving NIV, thus reducing asynchronies. It is of note that the association between ΔP_es_ and onset of AL was not significant. However, giving the significant rate of air leak in patients on HFNC, in which ΔP_es_ is the major determinant of ΔP_L_, the harmful effect of inspiratory effort in promoting alveolar rupture in this subset of patients at this disease stage remains matter of debate. Notwithstanding and considering the comparable values of ΔP_es_ between groups, the significant association found between AL onset and ΔP_L_ might suggest a role of NRS (rather than spontaneous breathing *per se*) in favoring PNX and/or PNM. Indeed, elevated PEEP and PS were found to be factors associated with AL only in patients receiving NIV, thus suggesting a potential role of NIV-induced VILI in this population. Experimental evidence showed how the combination between elevated inspiratory effort and ineffective pressure support, to produce an elevated ΔP_L_ value, can increase the risk of lung barotrauma and NIV failure (25, 26). Although respiratory drive might be independent from the level of support – especially in more severe patients –, our data further encourage to modulate respiratory support according to patient drive with the aim to reduce the patient’s effort (19).

The pathophysiology of COVID-19-associated acute lung injury is not limited to the alveolar damage induced by the inflammatory response (27). An impaired lung perfusion, sustained by impaired hypoxic vasoconstriction, pulmonary angiopathy and disseminated intravascular coagulation, are likely to evolve into the most severe pattern of this disease (1). These findings have been related to abnormal extracellular matrix degradation, vascular endothelial injury and lung interstitial tissue fragility (28). Overall, in COVID-19, the mortality rate rises when pulmonary damage is associated to an elevated D-dimer concentration (29); indeed in our study, patients developing AL had a high D-dimer level, suggesting a peculiar phenotype with a lung tissue prone to alveolar rupture if subject to high mechanical stress (23). The relative “fragility” of COVID-19 parenchyma has been suggested by Protti and coworkers in sedated-paralyzed mechanically ventilated patients, in whom air leak occurred with ventilator settings considered “protective” in other forms of ARDS (30). Furthermore, our data show that the consolidative lung impairment seen on CT was also significantly associated with AL. This might be related to the unfavorable interplay between lung inhomogeneity and asymmetrical forces distribution during inflation in the more advanced lung phenotypes of COVID-19 (1, 3, 26), and supports the speculation that barotrauma cannot be the only pathophysiological mechanism underlying lung injury in these patients.

Once more, our data confirm previous observations reporting that the occurrence of PNX-PNM under NRS assistance to treat the COVID-19 related ARF negatively impact clinical outcomes (31). Our patients who developed AL presented a 270% increase in the proportional risk of being dead or still under MV at day 28 as compared to patients who did not. Moreover, they had longer stay in RICU and worse 90-day mortality. Recent radiological studies have shown that the more critical forms of COVID-19 pneumonia may be associated with architectural distortion and micro-cysts formation (32) linking to more severe systemic inflammation with pulmonary microangiopathy (33). Overall elevated D-dimer, consolidative lung patterns, and a more intense ventilatory strategy as observed in the more severe forms of COVID-19 might increase the risk for PNX/PNM.

Our study has several limitations. First, the retrospective and single center design of the study, although powered by matching procedures, does affect the generalizability of results. Second, the limited number of patients does not allow sub-group analysis based on the different or combined types of AL. Third, the choice of ΔP_es_ and ΔP_L_ values used for analysis in NAL group was arbitrarily made (see the section “MATERIALS AND METHODS”), but not strictly related to any specific clinical stage or condition over the RICU stay. Moreover, the absence of information on expiratory tidal volume does not allow do draw firm conclusion on the role of NRS on VILI in this subset of patients. Additionally, the lack of blood and alveolar biomarkers (i.e., interleukin-6) does not allow definitive speculations about the role of systemic and local inflammation in promoting AL. Finally, the role of pressure support in promoting the onset of air leak need to be clarified with further investigation including matched patients undergoing invasive mechanical ventilation.

To conclude, higher values of dynamic transpulmonary pressure, blood D-dimer, pressures delivered during NIV, and a consolidative lung pattern were found to be significant risk factors for AL in spontaneously breathing COVID-19 patients with severe ARF which resulted in a worse prognosis. The application of NIV may play a role in promoting the occurrence of air leak(s) in severe COVID-19 patients with higher inflammation and vascular lung damage or remodeling.

## Data Availability Statement

The original contributions presented in the study are included in the article/Supplementary Material, further inquiries can be directed to the corresponding author.

## Ethics Statement

The studies involving human participants were reviewed and approved by the Area Vasta Emilia Nord. The patients/participants provided their written informed consent to participate in this study.

## Author Contributions

RT and GB equally contributed to the conception and design of the study, patients’ enrollment, data analysis, and article’s writing. LM, RF, LT, IC, FT, DA, FG, and MP made substantial contributions to the literature review, data collection, and manuscript writing. LD, SBa, EF, BB, and CM reviewed the literature, analyzed the data, wrote the manuscript, and produced the figures. MG, AM, and EC designed the study and reviewed and edited the manuscript. All authors have read and approved the final version of the manuscript.

## Conflict of Interest

The authors declare that the research was conducted in the absence of any commercial or financial relationships that could be construed as a potential conflict of interest.

## Publisher’s Note

All claims expressed in this article are solely those of the authors and do not necessarily represent those of their affiliated organizations, or those of the publisher, the editors and the reviewers. Any product that may be evaluated in this article, or claim that may be made by its manufacturer, is not guaranteed or endorsed by the publisher.

## Footnotes

1
https://www.who.int/emergencies/diseases/novel-coronavirus-2019
