# Supplementary material for: Risk Factors for Pulmonary Air Leak and Clinical Prognosis in Patients With COVID-19 Related Acute Respiratory Failure: A Retrospective Matched Control Study
Source: Front Med (Lausanne). 2022 Mar 31;9:848639. doi: 10.3389/fmed.2022.848639 (PMC9008271; doi:10.3389/fmed.2022.848639)
Supplement: Supplementary file 1 [file Table_1.DOCX]

**Supplementary materials**

**eTable 1. General and clinical features of the study population presented according to the type of respiratory support and the onset of air leak.**

|  | **HFNC cohort**  **n= 23** | |  | | **NIV cohort**  **= 33** | |  |
| --- | --- | --- | --- | --- | --- | --- | --- |
| **Variable** | ***Air leak (AL)***  ***n = 9*** | ***No air leak (NAL)***  ***n = 14*** | ***p value*** | | ***Air leak (AL)***  ***n = 19*** | ***No air leak (NAL)***  ***n = 14*** | ***p value*** |
|  |  |  |  | |  |  |  |
| **Age, years (IQR)** | 74 (59 – 79.5) | 68 (54 - 75) | 0.3 | | 74 (63 – 75) | 74 (71-77) | 0.7 |
| **Male sex, n (%)** | 6 (67) | 9 (64) | 0.9 | | 12 (63) | 10 (71) | 0.7 |
| **BMI, Kg/m^2^** | 24.5 (23 – 27.7) | 27 (24 – 30) | 0.2 | | 26.5 (25.4 – 28.7) | 27 (23 – 27) | 0.3 |
| **SOFA, score (IQR)** | 3 (3 – 4) | 3 (2 – 4) | 0.9 | | 3 (3 – 4) | 4 (3 – 4) | 0.5 |
| **Charlson index, score (IQR)** | 2 (2 – 5) | 2 (2 – 4) | 0.8 | | 2 (2 – 4) | 2 (1 – 5.5) | 0.9 |
| **PaO_2_/FIO_2_, mmHg (IQR)** | 102 (92 – 114) | 105 (84 – 137) | 0.7 | | 100 (88 – 120) | 101 (87 – 113) | 0.4 |
| **RR, bpm (IQR)** | 26 (24 – 30) | 25 (24 – 28) | 0.6 | | 28 (25.5 – 30.5) | 27 (24 – 29) | 0.6 |
| **MAP, mmHg (IQR)** | 77.5 (62.5 – 87.5) | 75 (68 – 86) | 0.7 | | 75 (65 – 90) | 80 (69 – 100) | 0.4 |
| **Lactate, mmol/L (IQR)** | 1.1 (0.8 - 1.5) | 1 (0.7 - 1.5) | 0.9 | | 1.2 (0.9 – 1.7) | 1 (0.6 - 1.4) | 0.6 |
| **Esophageal pressure swing, cmH_2_O (IQR)** | 12 (11 – 17) | 13.5 (10 – 18) | 0.7 | | 14 (11 – 18) | 12 (11 – 15) | 0.4 |
| **Dynamic transpulmonary pressure, cmH_2_O (IQR)** | 12 (11 – 17) | 12 (11 – 17) | 0.7 | | 24 (22 – 27) | 18 (15 – 22) | 0.02 |
| **Time from disease onset to RICU admission, days (IQR)** | 8 (5 – 13.5) | 10 (4 – 13) | 0.2 | | 6 (4 – 8) | 8 (4 – 10) | 0.5 |
| **Laboratory tests** |  |  |  | |  |  |  |
| White cells count, n*10^9^/L (IQR) | 7.5 (0.8 – 12.5)) | 5.4 (3.2 – 10) | 0.04 | | 6.7 (4.9 – 8.9) | 5.3 (3.1-9.8) | 0.8 |
| C-Reactive Protein, mg/dL (IQR) | 11.2 (8 – 18) | 8.7 (6 – 13.5) | 0.02 | | 12 (8 – 18) | 9.9 (5.2 – 16) | 0.5 |
| D-Dimer, µg/L (IQR) | 5467 (3440 – 16.843) | 1020 (858 – 1430) | <0.0001 | | 2130 (1200 – 8790) | 1200 (995 – 2520) | 0.04 |
| **Pharmacological treatment** |  |  |  | |  |  |  |
| Systemic steroids, n (%) | 9 (100) | 12 (86) | 0.5 | | 18 (95) | 10 (71) | 0.1 |
| Tocilizumab, n (%) | 7 (78) | 14 (100) | 0.1 | | 13 (68) | 14 (100) | 0.03 |
| **Non-invasive support pressure** |  |  |  |  |  |  |  |
| PEEP, cmH_2_O (IQR) | 6 (5 – 6) | 6 (5 – 6) | 0.7 | | 10 (8 – 10) | 8 (8 – 9) | 0.01 |
| PSV, cmH_2_O (IQR) | --- | --- | --- | | 10 (10 – 14) | 8 (8 – 10) | 0.01 |
| **Radiographic pattern** |  |  |  | |  |  |  |
| Interstitial, n (%) | 3 (33) | 8 (57) | 0.4 | | 6 (32) | 9 (64) | 0.08 |
| Consolidative, n (%) | 6 (67) | 6 (43) | 0.4 | | 13 (68) | 5 (36) | 0.08 |

Data are presented as number and percentage for dichotomous values or median and interquartile range (IQR) for continuous values.

**Abbreviations:** *IQR = Inter Quartile Range; RR = Respiratory Rate; MAP = Mean Arterial Pressure, SOFA = Subsequent Organ Failure Assessment score, HFNC = High Flow Nasal Cannula; NIV = Non-Invasive mechanical Ventilation; PEEP = Positive end expiratory pressure; PSV = Pressure support; LDH = Lactic dehydrogenase; BUN = blood urea nitrogen.*

**eFigure 1. Esophageal pressure swings waveform**

*
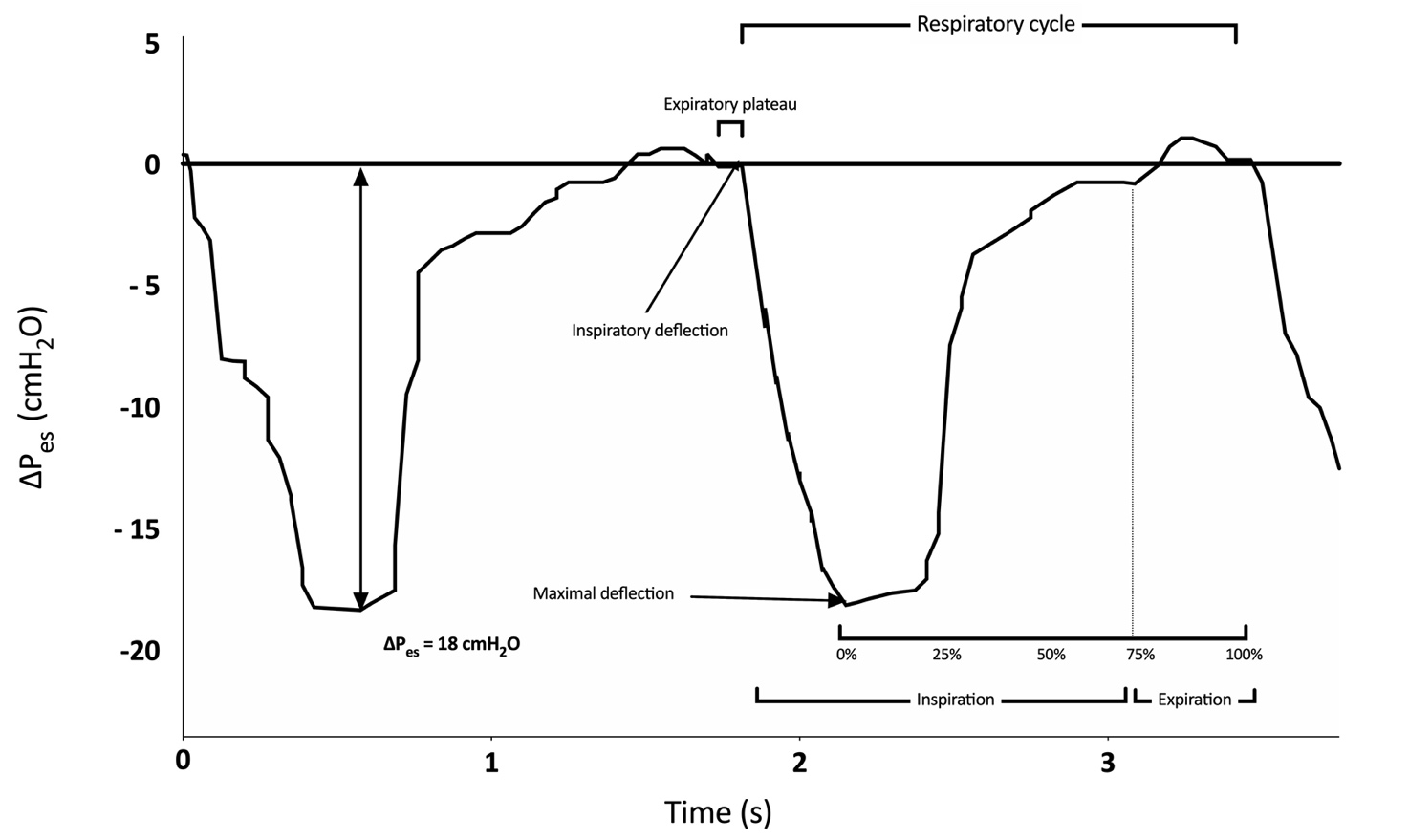
*

**eFigure 1.** Graphical representation ΔP_es_ swings waveform. The beginning of the inspiratory phase was identified at the time of P_es_ initial decay, while the end of inspiration was considered at the point of P_es_ that elapsed 25% of time from its maximum deflection to return to baseline.

**Abbreviations:** ΔP_es_ *= esophageal pressure*

**eFigure 2. Risk factors for air leak onset while on spontaneous breathing during COVID-19 acute respiratory failure according to NRS**

**eFigure 2.** Multiple logistic regression analysis showing the association between clinical, physiological and radiological variables with the occurrence of pneumothorax and pneumomediastinum in the study cohort by means of odds ratios (open diamonds) and relative 95% confidence intervals (error bars) in patients receiving HFNC (panel A) and NIV (panel B). For HFNC patients (panel A): D-dimer OR = 3.2 [2 – 8.5], C-reactive protein OR = 2.3 [1.1 – 4]. For NIV patients (panel B): dynamic transpulmonary pressure OR =2.6 [1.4 – 5.4], PEEP OR = 3.2 [2 - 6.5], PSV = 3.5 [1.8 – 7.2]. * p=0.02, **p=0.03, ***p=0.04. Significance was set for p<0.05.

*CT, computed tomography; PSV, pressure support; PEEP, positive end-expiratory pressure; NIV, non-invasive ventilation; HFNC, high-flow nasal cannula; RICU, respiratory intensive care unit; MAP, mean arterial pressure; RR, respiratory rate; SOFA sequential organ failure assessment; BMI, body mass index.*
